# A Study on a High-Precision 3D Position Estimation Technique Using Only an IMU in a GNSS Shadow Zone

**DOI:** 10.3390/s25237133

**Published:** 2025-11-22

**Authors:** Yanyun Ding, Yunsik Kim, Hunkee Kim

**Affiliations:** AI System Design Lab, Department of Advanced Materials Processing Engineering, Inha University, 36 Gaetbeol-ro, Yeonsu-gu, Incheon 21999, Republic of Korea; dyy000720@inha.edu (Y.D.); yskim9701@inha.edu (Y.K.)

**Keywords:** localization, human detection and tracking, GPS shadow zone, inertial measurement unit, pedestrian dead reckoning (PDR)

## Abstract

**Highlights:**

**What are the main findings?**

- A novel 3D trajectory estimation mechanism was developed using a single 9-axis IMU that integrates stride-length estimation, heading alignment, and stair-state recognition, enabling accurate and continuous tracking without external references in GNSS-denied environments.
- Experiments on straight paths, turning routes, and multi-floor stairs showed high accuracy: horizontal error remained below 3% in 100 m, vertical displacement error stayed within 2%, and heading deviation stayed at a sub-radian level, demonstrating excellent stability.

**What is the implication of the main finding?**

- The proposed mechanism provides a practical and flexible solution for precise agent trajectory estimation in indoor or GNSS-shielded environments, even under resource-constrained conditions.
- Its accuracy and robustness across mixed gait patterns and vertical transitions support applications such as indoor navigation, emergency response, underground inspection, and wearable/embedded positioning systems.

**Abstract:**

In Global Navigation Satellite System (GNSS)-denied environments, reconstructing three dimensional trajectories using only an Inertial Measurement Unit faces challenges such as heading drift, stride error accumulation, and gait recognition uncertainty. This paper proposes a path estimation method with a nine-axis inertial sensor that continuously and accurately estimates an agent’s path without external support. The method detects stationary states and halts updates to suppress error propagation. During motion, gait modes including flat walking, stair ascent, and stair descent are classified using vertical acceleration with dynamic thresholds. Vertical displacement is estimated by combining gait pattern and posture angle during stair traversal, while planar displacement is updated through adaptive stride length adjustment based on gait cycle and movement magnitude. Heading is derived from the attitude matrix aligned with magnetic north, enabling projection of displacements onto a unified frame. Experiments show planar errors below three percent for one-hundred-meter paths and vertical errors under two percent in stair environments up to ten stories, with stable heading maintained. Overall, the method achieves reliable gait recognition and continuous three-dimensional trajectory reconstruction with low computational cost, using only a single inertial sensor and no additional devices.

## 1. Introduction

In dynamic real-world environments, positioning signals from Global Navigation Satellite Systems (GNSSs) are highly susceptible to blockage or interference. This issue is particularly pronounced in typical non-line-of-sight (NLOS) scenarios, including urban canyons, underground passageways, the interiors of high-rise buildings, and densely forested areas. Such settings are generally categorized as GNSS-denied environments, where satellite-dependent navigation systems often become inoperative, leading to a dramatic increase in position estimation uncertainty. In conventional navigation systems, IMU measurements are often integrated to predict trajectories, while GNSS observations are employed to correct drift and maintain long-term stability [1,2,3,4]. However, GNSS signals are not only unavailable in many constrained environments but are also vulnerable to intentional interference, such as jamming and spoofing [5]. Many studies have attempted to design alternative frameworks to achieve robust trajectory estimation in GNSS-denied environments [6,7,8]. Representative examples include research on unmanned aerial vehicle navigation using SLAM and path planning in GNSS-denied environments [9], studies on hybrid UWB/vision/inertial fusion systems [10], and IMU-based deep learning approaches for trajectory estimation [11]. Consequently, the development of 3D position estimation and trajectory reconstruction techniques that maintain high accuracy and robustness under limited or absent GNSS support has remained a persistent research challenge within the domains of mobile perception and spatial intelligence.

**Figure 1:**
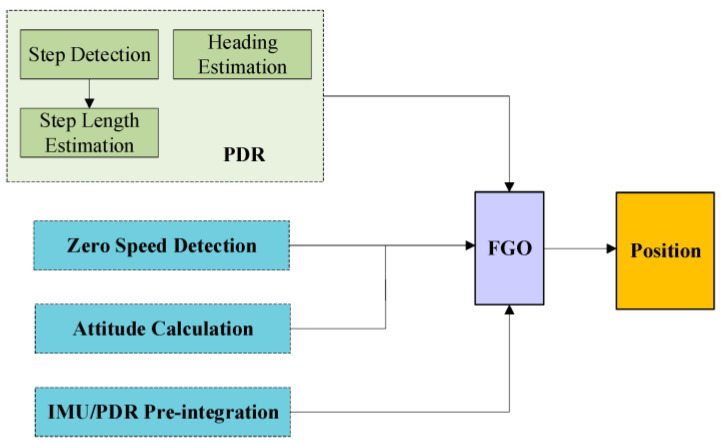
Framework diagram of pedestrian positioning algorithm based on FGO [25].

To address these challenges, both academic and industrial communities have extensively explored multi-sensor fusion frameworks. Representative systems typically integrate various heterogeneous sensors, such as LIDAR [12], camera [13], RSSI [14], ultra-wideband modules (UWB) [15,16,17], and radar [18,19], with an inertial measurement unit (IMU) serving as the core sensing component. These systems commonly adopt estimation techniques such as the Extended Kalman Filter (EKF) [20] or Factor Graph Optimization (FGO) [21] to achieve real-time position estimation and spatial structure reconstruction, even under dynamic and complex environmental conditions. Such sensor fusion strategies have yielded notable improvements in localization accuracy and robustness across a range of real-world scenarios. For instance, a radar-beacon-based wireless localization system (WLPS) has demonstrated centimeter-level precision in autonomous drone landing operations [22], while simultaneous localization and mapping (SLAM) systems combining GNSS and LiDAR have proven effective for large-scale map generation and high-precision navigation in challenging terrains [23]. More recently, FGO-based approaches that incorporate GNSS signals with pedestrian dead reckoning (PDR) and road anchor constraints [24], as well as dynamic stride length estimation models based on a hybrid Transformer and Temporal Convolutional Network (TCN) architecture [25], have shown superior positioning stability compared to conventional EKF-based methods. Similarly, WiFi/PDR integrated navigation schemes employing constraint-based Kalman filters have also been reported [26], demonstrating improved positioning accuracy in GNSS-constrained environments. To further clarify the proposed pedestrian localization framework, Figure 1 illustrates how the dynamic step length estimation model, constructed by integrating Transformer and TCN architectures, operates within the overall algorithm. The framework is composed of gait detection, Transformer–TCN-based step length estimation, heading estimation, IMU/PDR pre-integration, and stationary state detection modules, with their outputs jointly processed through factor graph optimization (FGO) to yield the final position estimate. This design emphasizes the pivotal role of dynamic step length estimation in enhancing trajectory reconstruction, while also demonstrating the capability of FGO to impose trajectory constraints and thereby improve localization accuracy under complex environmental conditions. Nevertheless, the practicality of many existing approaches still depends on GNSS aiding signals, which leads to significant performance degradation in fully indoor or GNSS-denied environments.

Although various sensor fusion-based positioning techniques have achieved impressive performance in controlled experimental settings, their applicability in real-world environments often falls short of expectations. Such systems generally require the installation of multiple external sensors and frequent synchronization, involve complicated calibration procedures, and incur very high maintenance costs [27,28]. Moreover, these sensors are highly sensitive to environmental conditions. Camera-based approaches are significantly affected by illumination, and their accuracy deteriorates markedly in dark scenes or under strong backlighting. While UWB-based indoor positioning systems [29] provide high ranging precision and strong resistance to multipath interference, their real-world implementation remains limited by anchor deployment density and strict synchronization requirements. Performance degradation under non-line-of-sight (NLOS) conditions and the high cost of infrastructure maintenance continue to restrict scalability in large or dynamically changing environments. In contrast, hybrid Bluetooth Low Energy (BLE) localization methods [30] that integrate Received Signal Strength Indicator (RSSI) and Angle-of-Arrival (AOA) measurements have shown improved robustness through adaptive confidence evaluation mechanisms.

Nevertheless, the achieved positioning accuracy typically remains at the meter level, and system performance still fluctuates significantly under signal attenuation or interference, indicating the inherent instability of radio-based measurements. Similarly, hybrid approaches that integrate Wi-Fi fingerprinting with pedestrian dead reckoning (PDR) face critical limitations due to the substantial cost associated with constructing and maintaining fingerprint databases [31]. Notably, infrastructure-dependent localization systems are not only expensive in terms of deployment and upkeep, but also require periodic recalibration whenever the surrounding environment changes, thereby compromising real-time applicability and long-term scalability. Furthermore, implementing complex optimization algorithms and achieving precise synchronization remains challenging on embedded platforms with constrained computational resources.

**Figure 2:**
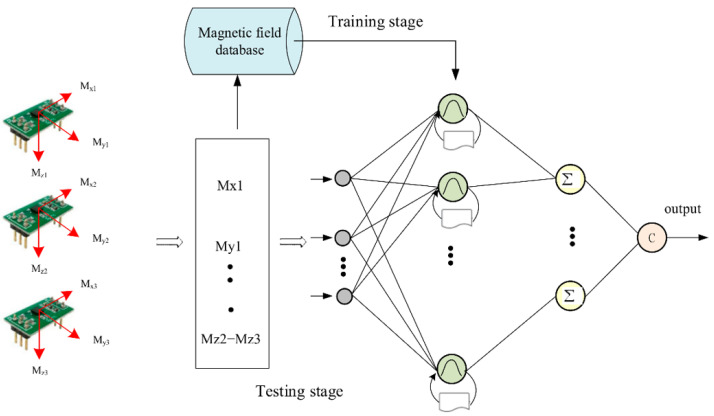
RPNN fingerprint map classifier (RFMC) [32]. Gray circles denote input nodes corresponding to tri-axial magnetic field measurements, green circles indicate neurons in the hidden layers, the icons below each green node denote groups of probabilistic neurons in the hidden layer that process the input magnetic features before the summation units, yellow circles (Σ) represent summation units, and arrows show the information flow from inputs to the final output class c.

Magnetic field–based indoor localization approaches have also been actively explored, including temporal convolutional network (TCN) models for magnetic time-series analysis and recurrent probabilistic neural network (RPNN)–based magnetic fingerprint mapping frameworks [32]. The RPNN-based magnetic fingerprint mapping classifier (RFMC), illustrated in Figure 2, extracts discriminative features from input magnetic signals using a neural network trained on a magnetic field database and subsequently classifies newly collected signals during testing to estimate position. Despite these advances, magnetic field-based methods remain highly sensitive to environmental variations and device heterogeneity, which restrict their long-term stability and cross-device generalization. Similarly, vision- and light-based localization techniques have been developed to complement magnetic and inertial approaches. Abdalmajeed et al. [33] proposed a machine-learning-based visible-light positioning system using LED light sources and geometric constraints to enhance spatial accuracy in various indoor settings. In parallel, Shao et al. [34] demonstrated a deep-learning-based vision inspection framework for automated object detection and counting, which addresses limitations of conventional imaging methods that are vulnerable to rotation, scale, and illumination changes. Nevertheless, the practical applicability of these optical methods remains restricted under dynamic lighting conditions or occlusions.

However, these vision-based methods remain strongly dependent on lighting conditions and are susceptible to occlusion. Therefore, although multi-sensor fusion and vision-based solutions can deliver high accuracy under controlled environments, their adaptability and practicality degrade significantly in real-world scenarios, including GNSS-denied spaces, dynamic environments, and computationally constrained platforms. These limitations have motivated both academia and industry to reorient research efforts toward low-cost, single-sensor, and high-accuracy localization strategies, with IMU-only approaches in particular gaining increasing attention.

Inertial navigation systems based on pedestrian dead reckoning (PDR) and zero-velocity update (ZUPT) are regarded as representative classical approaches under single inertial measurement unit (IMU)-only conditions [35]. PDR employs step frequency and stride-length models to achieve stable short-term trajectory estimation, whereas ZUPT mitigates velocity drift during walking halts through stationary state detection [36]. Although these methods provide the benefits of structural simplicity and high computational efficiency, their effectiveness is constrained by dependencies on positional accuracy, gait recognition, and pose estimation precision. As a result, they remain highly susceptible to IMU noise and bias drift, and face difficulties in sustaining reliable performance in complex or long-duration environments.

**Figure 3:**
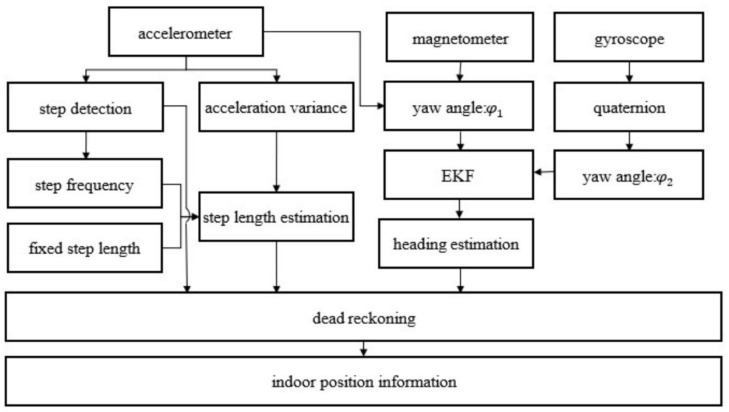
PDR structure frame diagram [37].

To address these limitations, several improvement strategies have been proposed. HUANG et al. [37] integrated a fixed-stride model with a linear-stride model through a weighted scheme, incorporating walking frequency and acceleration variance as features to simultaneously preserve the stability of the fixed model and the flexibility of the linear model. This approach achieved a position estimation accuracy of 98.47% on a 42.6 m straight path. The structure of the pedestrian navigation system proposed in this study is shown in Figure 3. LI et al. [38] combined gait pattern recognition with adaptive stride estimation to substantially enhance 3D trajectory reconstruction accuracy, reducing the average error to 1.09 m. Nevertheless, despite their strong short-term performance, these methods remain constrained by parameter dependency, limited adaptability, and high computational complexity.

**Figure 4:**
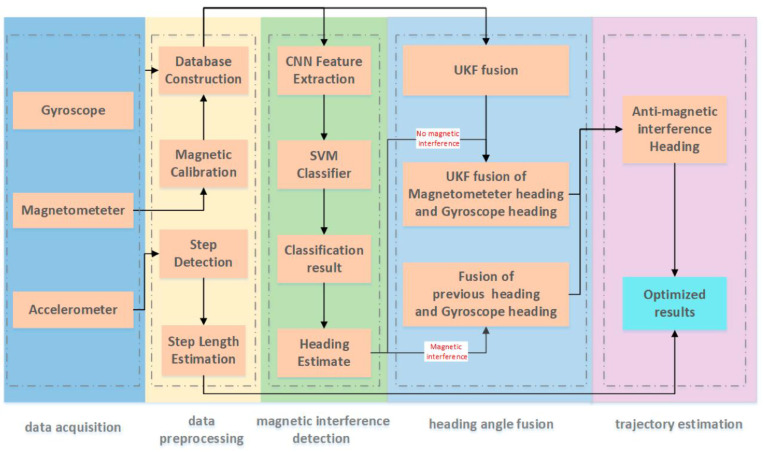
Framework of the heading estimation algorithm based on CNN–SVM for magnetic interference detection [42].

To further enhance system performance, researchers have explored the integration of deep learning techniques for processing IMU time-series data. Representative examples include ORINET [39], a pose estimation framework that leverages long short-term memory (LSTM) neural networks in combination with a dynamic dropout strategy, and a lightweight navigation system that recovers orientation using a convolutional neural network (CNN) [40]. For instance, LEE et al. [41] proposed a method in which feature sequences extracted from a CNN are fed into a gated recurrent unit (GRU) network, while a Kalman filter is incorporated to suppress multi-scale errors. Their experiments demonstrated the effectiveness of this approach in GPS-shadowed environments. In addition, a CNN–SVM-based magnetic interference compensation method has been proposed [42], and its overall architecture is illustrated in Figure 4. As shown, the system acquires data from accelerometer, gyroscope, and magnetometer sensors, and detects magnetic interference using CNN-based feature extraction followed by SVM classification after preprocessing. When interference is absent, the heading is estimated through UKF fusion of gyroscope and magnetometer data; when interference is present, the heading is corrected by combining gyroscope outputs with previous estimates. Finally, the compensated heading estimate is generated and applied to trajectory reconstruction.

Although these methods have demonstrated strong performance on specific benchmark datasets, they encounter several challenges in real-world applications. First, the reliance on large volumes of scene-specific training data necessitates repeated data collection and retraining when transferred to new environments, thereby prolonging deployment cycles and hindering rapid adaptation. Second, their architectural complexity, sensitivity to hyperparameters, difficulty of model tuning, and limited interpretability impede clear technical understanding of performance deviations. Third, the computationally intensive nature of inference restricts practical deployment on resource-constrained devices. Most critically, these purely data-driven approaches lack physical motion constraints and effective integration with environment-specific prior knowledge, which results in the accumulation of estimation errors and unstable trajectory reconstruction in unstructured environments.

Building upon these prevailing research directions, this study develops an IMU-only 3D path reconstruction system that eliminates the need for learning models, ensures broad applicability, and enables flexible deployment in diverse field environments. The central concept of the proposed system lies in integrating physics-based motion modeling with a time-series state recognition mechanism. This design enhances adaptability to environmental variations at the sensing layer, while structurally guaranteeing both deployment simplicity and real-time computational efficiency.

In this study, we design a mechanism that leverages a single IMU sensor and the extrema of *Z*-axis acceleration to capture vertical movement rhythms and reliably distinguish three gait states—flat ground (FLAT), stair ascent (UP_STAIRS), and stair descent (DOWN_STAIRS)—without requiring external compensation or map assistance. Stride length is subsequently estimated in an adaptive manner using gait cycle and acceleration amplitude information, while trajectory updates are performed with respect to the X^+^ direction, defined as the reference axis of the motion vector within the posture matrix. This enables a 3D path reconstruction process that preserves heading direction while dynamically adjusting stride length according to walking conditions. Furthermore, the system incorporates a stationarity detection function that suspends trajectory computation when motion ceases or speed decreases, thereby effectively mitigating the accumulation of integration errors.

The method proposed in this study exhibits several distinctive advantages. First, it operates independently of external calibration or environment-specific learning models, thereby ensuring strong adaptability across diverse settings. In addition, its structurally concise design guarantees high real-time performance and computational efficiency, rendering it well-suited for portable devices and edge computing platforms. Moreover, the incorporation of a closed-loop feedback framework—where state recognition and position inference dynamically influence one another—significantly strengthens the system’s capacity to robustly identify movements, even under conditions of frequent transitions or abrupt actions.

However, due to the nonlinear error characteristics inherent in IMUs and the accumulation of sensor drift over time, accuracy can degrade in long-term and highly dynamic environments. To address these challenges, further refinement of stationary-state correction techniques, the incorporation of environmental geometric constraints, and the development of extended models capable of adapting to diverse gait patterns are required. These research directions will complement the limitations of the IMU-only approach proposed in this study and play a critical role in enhancing its applicability to long-duration operations and complex real-world scenarios.

Building upon the above literature, it can be observed that prior IMU-based pedestrian localization studies generally fall into two distinct categories. The first focuses on planar displacement estimation, employing stride-length modeling and zero-velocity updates (ZUPT) to reconstruct horizontal trajectories with high short-term accuracy. The second addresses vertical motion recognition, extracting peak–valley rhythms from *Z*-axis acceleration to classify stair ascent and descent. However, these two directions have traditionally been investigated independently, which leads to trajectory discontinuities and accuracy degradation when pedestrians transition between flat walking and stair climbing.

The present study introduces a unified, physics-driven 3D reconstruction mechanism that couples adaptive stride-length estimation with rhythm-based three-state recognition within a quaternion-defined posture framework. This integration allows the system to maintain geometric and temporal continuity across both planar and vertical movements using only a single IMU, without relying on external references or learning models. To the best of our review, no previous IMU-only work has established such an integrated mechanism that jointly handles planar and vertical gait dynamics within one coordinate-consistent process. The proposed approach therefore represents a novel structural paradigm for lightweight, learning-free, and continuous 3D trajectory reconstruction in GNSS-denied environments

## 2. Materials and Methods

The research method presented in this chapter is designed to address the challenge of 3D trajectory reconstruction based on gait information in environments where satellite navigation signals are unavailable. The system mechanism leverages the temporal continuity of inertial measurement data and the periodic characteristics of human gait, and is composed of five core functional modules that operate in an integrated manner. Specifically, it incorporates a module for acquiring and preprocessing multidimensional IMU data, a mechanism for promptly detecting user stationarity and halting integral computations, a function for discriminating three-phase locomotion modes through *Z*-axis rhythmic analysis, a model for estimating step length by coupling gait cycle with acceleration magnitude, and, finally, a procedure for generating a 3D motion trajectory using posture information.

**Figure 5:**
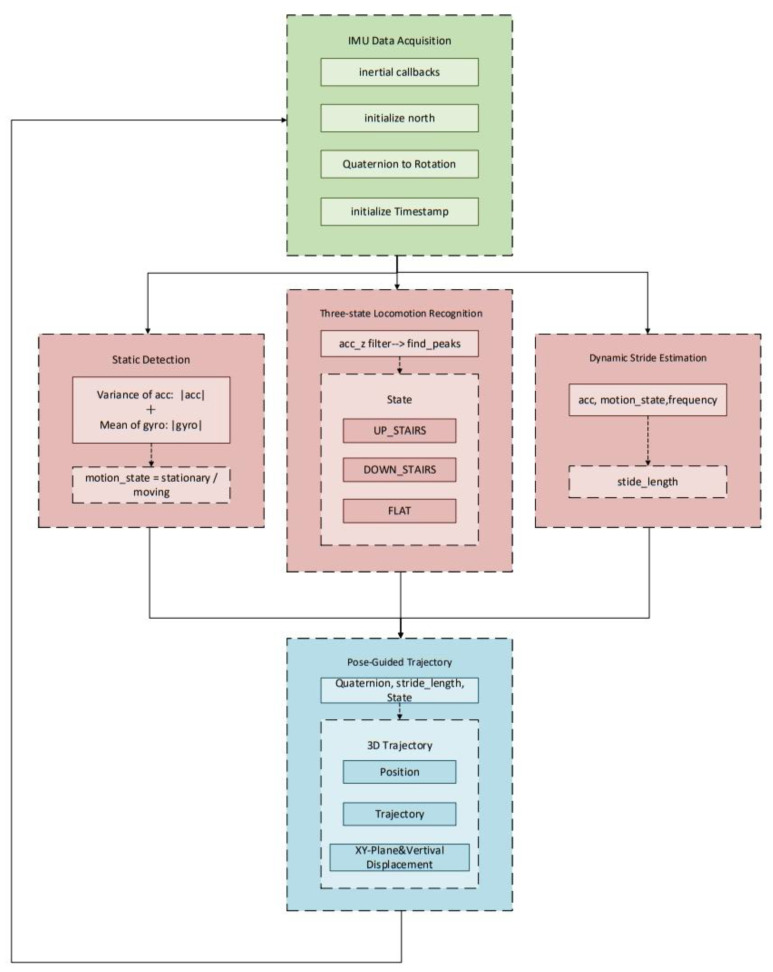
Overall System Architecture. Arrows indicate the data flow between modules.

The entire system operates in a dataflow-oriented manner, sequentially adhering to the callback cycle of inertial measurements. Sensor data are first acquired and subsequently propagated through successive stages including state recognition, stride estimation, and posture analysis, ultimately enabling the 3D reconstruction of the movement trajectory within a spatial coordinate mechanism. Figure 5 depicts the system architecture and information flow, providing a visual representation of the functional interconnections among modules and the complete process from signal acquisition and recognition to spatial reconstruction.

### 2.1. IMU Multi-Sensor Data Collection and Initialization

**Figure 6:**
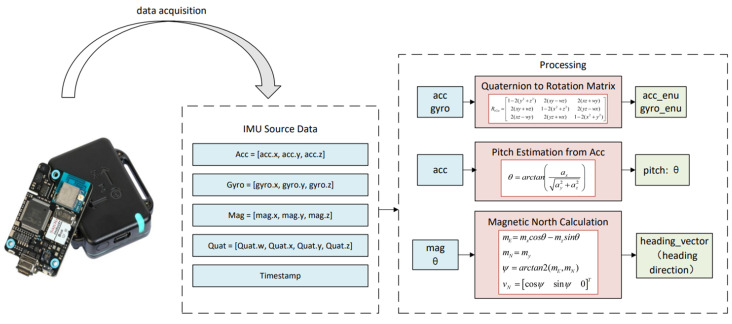
Data collection and processing process.

To establish a reliable foundation for subsequent gait-state recognition and trajectory estimation, this study first performs multi-sensor data acquisition and initialization using the X-IMU3 module [43]. The inertial measurement unit simultaneously records raw acceleration, angular velocity, magnetic field, and quaternion orientation data at synchronized timestamps. Figure 6 illustrates the overall workflow of this acquisition and preprocessing process, highlighting how multi-sensor signals are organized, converted, and prepared for subsequent orientation inference.

To ensure stability and geometric consistency in spatial orientation representation, the system is designed to continuously acquire and interpret multi-sensor data for 3D path reconstruction, with the X-IMU3 inertial measurement unit (IMU) serving as the core component [44].

The raw sensor outputs include tri-axial acceleration, tri-axial angular velocity, tri-axial magnetic field strength, and attitude quaternions. These data streams are reconstructed according to a unified time index and consolidated into a single data array. This structure not only ensures accurate time-domain fusion of heterogeneous sensor information but also establishes the foundational mechanism for delivering orientation information in a consistent and traceable manner throughout subsequent attitude inference and orientation computation.

To realize distortion-free transformation from the body coordinate system to the geographic coordinate system, the system employs a quaternion-based rotation matrix as an intermediate structure for coordinate projection. This rotation matrix is analytically derived from the unit quaternion $q={\left[w,x,y,z\right]}^{T}$, and its formulation is expressed as follows:

(1) $$R_{b2n}=\left[\begin{array}{ccc} 1-2(y^{2}+z^{2}) & 2(xy-wz) & 2(xz+wy)\\ 2(xy+wz) & 1-2(x^{2}+z^{2}) & 2(yz-wx)\\ 2(xz-wy) & 2(yz+wx) & 1-2(x^{2}+y^{2}) \end{array}\right]$$

This rotation matrix exhibits strict orthogonality and a unit determinant, thereby preserving vector magnitudes and relative angles during coordinate transformations. Based on this matrix, the acceleration $a_{b}$ and angular velocity $\omega_{b}$ measured in each frame are mapped into the geographic coordinate system:

(2) $$a_{n}=R_{b2n}\times a_{b},\;\omega_{n}=R_{b2n}\times \omega_{b}$$

To ensure that the system’s initial orientation is physically valid and consistent with the spatial reference frame, a geomagnetism-based initialization mechanism is employed to estimate the initial planar heading using the geomagnetic azimuth. Specifically, magnetometer data are projected and corrected onto the horizontal plane with the aid of the pitch angle θ estimated from the acceleration component. The resulting projection vector is computed as follows:

(3) $$\begin{array}{l} m_{E}=m_{x}cos\theta -m_{z}sin\theta \\ m_{N}=m_{y} \end{array}$$

From this projection, the navigation heading angle ψ can be derived, and its calculation is given as follows:

(4) $$\psi =arctan2(m_{E},m_{N})$$

Based on this, the unit direction vector in the horizontal plane is defined as follows:

(5) $$v_{N}={\left[\cos\psi \;\sin\psi \;0\right]}^{T}$$

To mitigate the influence of geomagnetic deviation and attitude drift during the initial orientation phase, the system collects a fixed interval of stable data at startup. Acceleration signals obtained over successive time windows are processed through gravity component separation and low-pass filtering to extract an average vector that reflects the true direction of movement. This vector is subsequently transformed into the geographic coordinate system via an initial rotation matrix $v_{N}$, which is then fine-tuned by comparison with the previously derived orientation reference, thereby ensuring that the reconstructed spatial orientation more accurately aligns with the actual trajectory. This procedure not only enhances the physical consistency of orientation definition but also facilitates the establishment of a cyclic orientation update mechanism during system initialization.

After this process, the data processing module outputs a calibrated rotation matrix, acceleration and angular velocity expressed in the geographic coordinate system, an aligned initial orientation reference, a sliding-window continuous inertial data sequence, and a computed pose quaternion. These outputs serve as inputs to subsequent modules—such as stationary state detection, locomotion mode recognition, and gait modeling—thereby supplying stable, low-drift reference information for the entire system.

### 2.2. Stationary State Detection and State Freezing Mechanism

To mitigate trajectory drift caused by accumulated integration errors during long-term motion, the system employs a stationarity detection mechanism based on inertial observations [45], together with a state-freezing strategy that suppresses redundant computations during invalid motion segments. The mechanism exploits the statistical stability of acceleration and angular velocity over short time windows and applies composite constraints derived from their standard deviations, thereby ensuring reliable recognition of stationary states while preserving sensitivity.

The system maintains a sliding observation window of length *N*, updating the standard deviations of acceleration and angular velocity at each cycle. If both metrics simultaneously satisfy the following criteria, the corresponding segment is classified as stationary:

(6) std(∥a∥)<δa,std(∥ω∥)<δω

Here, δa and δω denote experimentally determined thresholds, and g presents the gravitational acceleration constant. When these conditions are satisfied, the system classifies the current state as stationary.

In the stationary state, the integration of velocity and displacement is immediately suspended to prevent error accumulation. Upon detecting a transition from stationary to moving, the system executes a reference step-length update mechanism and resamples the current posture rotation matrix, thereby ensuring continuity and consistency in orientation and scale estimation at the onset of motion. The stationarity detection logic operates continuously at each inertial data update cycle, outputting a Boolean signal, current_motion_state. This signal is jointly referenced by core modules such as path reconstruction, pose estimation, and gait modeling, and functions as a global switch in the system-wide motion control logic. Through this mechanism, integration drift during low-speed phases is effectively suppressed, and the robustness and drift resistance of trajectory estimation are significantly enhanced, even under long-term motion.

### 2.3. Movement State Recognition and Gait Modeling

**Figure 7:**
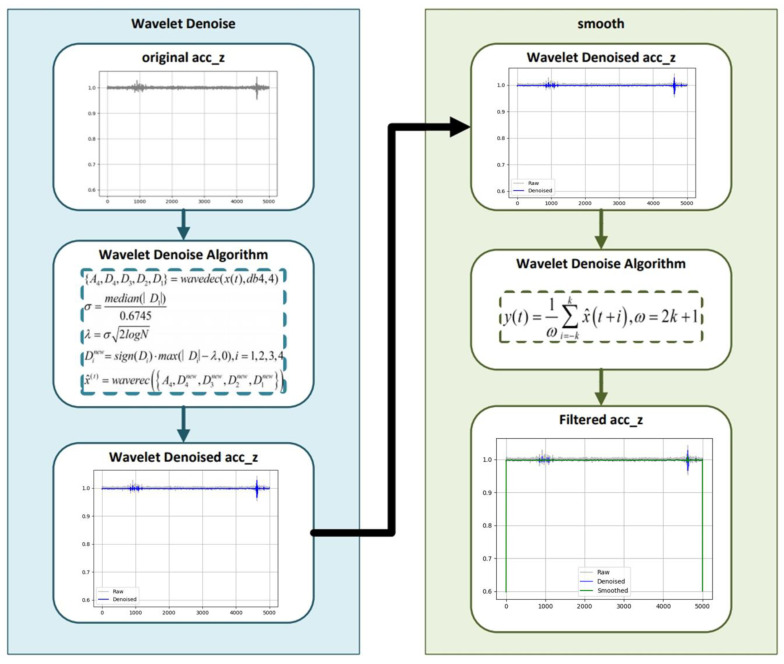
Acc_z filter.

To ensure accurate recognition of motion states from IMU signals, it is necessary to suppress high-frequency noise and restore the intrinsic periodicity of the acceleration waveform prior to gait modeling. In this study, the *Z*-axis acceleration signal, which contains the most distinct rhythmic components of walking motion, is selected for analysis. The preprocessing stage consists of a two-step filtering strategy combining wavelet-based denoising and moving-average smoothing. Figure 7 illustrates the structure of this filtering process and its effect on the *Z*-axis acceleration signal, providing the foundation for reliable gait-state segmentation and the subsequent three-state recognition mechanism [1,2,3,4,5,6].

During walking, *Z*-axis acceleration signals exhibit regular repetitive patterns and contain modal information, with these motion characteristics becoming particularly pronounced during stair ascent and descent. Accordingly, the system implements a three-state recognition method based on *Z*-axis extrema rhythms, enabling reliable discrimination among flat walking (FLAT), stair climbing (UP_STAIRS), and stair descending (DOWN_STAIRS) through structural analysis of the acceleration signals. To achieve this, wavelet-based denoising [46] is first applied to the raw *Z*-axis acceleration signals, followed by a moving-average filter to suppress residual noise and sensor disturbances.

This two-stage filtering not only enhances the periodic structure of gait signals but also mitigates the effects of IMU measurement noise, bias drift, and external vibration interference, thereby improving the stability of state recognition across different environments and sensor conditions.

**Figure 8:**
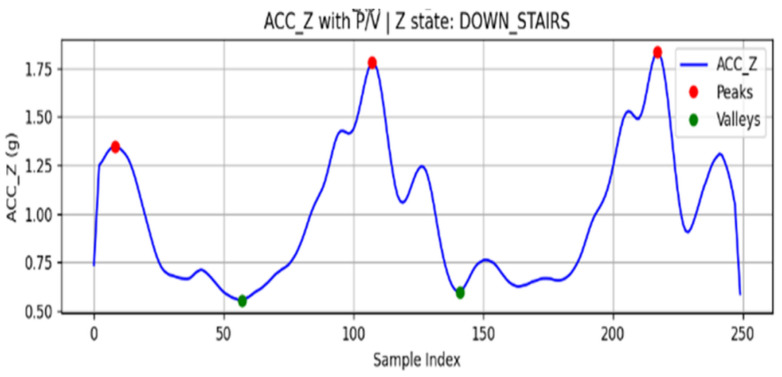
ACC_Z extreme value search results.

Local maxima (P and minima (V) are then extracted as candidate feature points [47], as shown in Figure 8. To avoid false detections caused by weak vibrations, a thresholding criterion is introduced to select the final feature points:

(7) P>1.2g, V<0.8g

Here, g is the gravitational acceleration constant.

When a sequence of cross-extreme structures that satisfy the threshold conditions is continuously detected, the system segments the signals into rhythmic units. Based on the extremum array patterns and temporal threshold conditions, the movement state is determined as follows:

- P–V–P structure: classified as stair climbing (UP_STAIRS).
- V–P–V structure: classified as stair descending (DOWN_STAIRS).
- If the extrema fail to meet the threshold conditions or the cross-extreme sequence is interrupted, the state is classified as flat walking (FLAT).

To ensure stability and robustness during state transitions, the system incorporates a buffer window together with an error-tolerance strategy in the state update mechanism. This design effectively eliminates misclassifications caused by transient interference or irregular gait patterns.

### 2.4. Dynamic Stride Estimation Model

**Figure 9:**
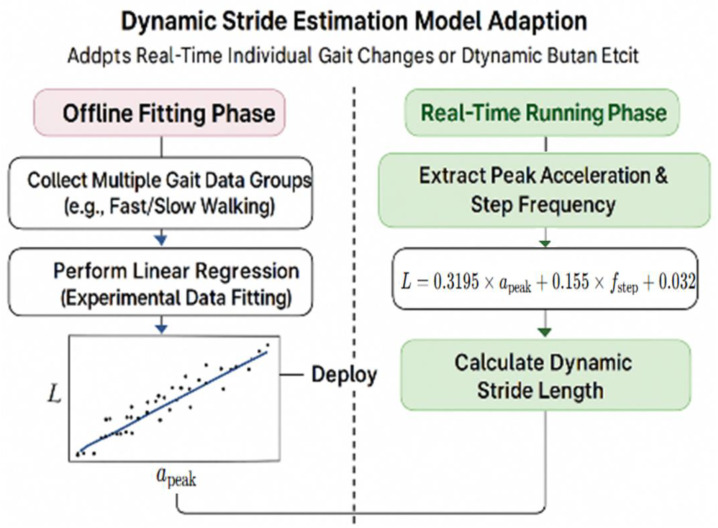
Adapting a dynamic stride estimation model. The black scatter points represent multiple gait samples collected in the offline phase, where each point corresponds to a data pair of peak acceleration peak apeak and measured stride length L used for linear regression fitting.

Accurate estimation of stride length is essential for maintaining scale consistency and improving the spatial accuracy of IMU-based pedestrian trajectory reconstruction. Variations in walking speed, step frequency, and individual gait characteristics can lead to non-negligible deviations if a fixed stride model is applied. To address this issue, the proposed system introduces a dynamic stride estimation mechanism that continuously adapts to motion intensity in real time. Figure 9 presents the conceptual framework of this model, illustrating the process of data collection, feature extraction, and the establishment of a linear stride-length prediction model based on acceleration peak and gait frequency.

To capture variations in individual gait characteristics in real time, the system employs a step-length estimation model that leverages acceleration peak values and walking frequency. The model adopts a two-stage strategy consisting of offline learning and online application. During the experimental phase, gait data—including triaxial acceleration, actual step length, and walking frequency—were collected. From each walking segment, the primary acceleration peak $a_{peak}$ and walking frequency f were extracted to construct a learning sample set $\left(a_{peak},f\right)\to L_{true}$. Subsequently, the least-squares method was applied to establish a linear model for step-length prediction:

(8) $$L=\alpha \cdot a_{peak}+\beta \cdot f+\gamma$$

Here, L denotes the estimated stride length, while α, β and γ are regression coefficients derived from experimental data. In this study, the following coefficients (α=0.3195, β=0.155, γ=0.032) were applied, reflecting the dominant contribution of acceleration peaks to stride length, the compensatory effect of gait frequency variations, and the individual’s baseline stride length.

The regression parameters were calibrated using experimental gait data from multiple participants, which minimizes individual walking-style bias and improves the model’s generalization capability across users and walking speeds.

To evaluate the engineering practicality of the proposed stride-length estimation model, a performance assessment was conducted using the Benchmark dataset [47]. This dataset contains over 10,000 stride samples collected from multiple users across indoor and outdoor walking scenarios, including stair ascent and descent, providing diverse gait patterns for offline evaluation.

The assessment focused on the statistical accuracy of stride-length prediction using standard error metrics—Maximum Absolute Error (MaxAE), Mean Absolute Error (MAE), and Root Mean Square Error (RMSE)—which, respectively, characterize the peak deviation, mean deviation, and overall dispersion of the estimation error. 

The results were as follows:

- MaxAE ≈ 9.2 cm;
- MAE ≈ 5.5 cm;
- RMSE ≈ 9.5 cm.

These values confirm that the proposed model achieves high accuracy and robustness under different gait conditions. It should be noted that this evaluation was performed offline to validate the mathematical model itself. In the subsequent experiments described in Section 3, this stride-length estimation module was embedded into the complete single-IMU 3D trajectory reconstruction framework and executed in real time to generate step-wise displacements online.

The analysis demonstrated excellent stability and validity across inter-individual differences and diverse walking conditions, effectively constraining single-stride length errors to within 6 cm. This value represents the local per-step deviation and remains consistent with the global positioning accuracy of below 3% observed on a 100 m horizontal trajectory. Because step-wise errors fluctuate around zero and tend to partially cancel each other during periodic gait cycles, and because the system applies stationarity-freezing only during true stationary intervals together with attitude-coupled displacement projection, long-term drift is effectively constrained and error accumulation avoided. Moreover, the model’s concise structure makes it well-suited for embedded systems and provides strong practical applicability by satisfying the accuracy and robustness requirements for real-world 3D path reconstruction.

In practical applications, the system extracts acceleration peaks $a_{peak}$ and gait frequencies f in real time for each rhythmic interval and directly applies them to the previously derived empirical stride estimation formula to compute the current stride length $L_{step}(t)$. Specifically, during the initial transition from rest to motion, the baseline stride length $L_{0}$ is recalibrated, while in subsequent continuous gait tracking, each stride length $L_{step}(t)$ is updated in real time. This design enhances the personalization and temporal consistency of path estimation, ensuring that the system maintains high robustness and practicality across a wide range of walking speeds and user conditions.

### 2.5. Three-Dimensional Path Reconstruction Based on Pose Combination

**Figure 10:**
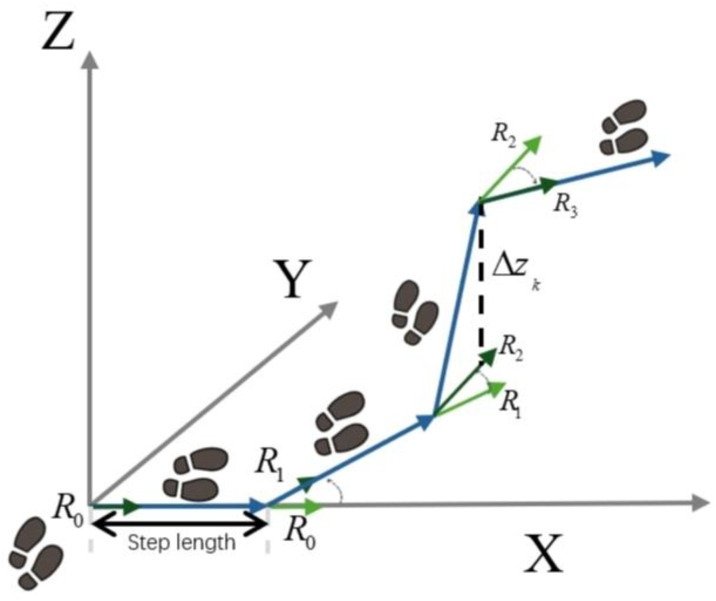
Path reconstruction concept diagram.

To achieve reliable three-dimensional positioning in GNSS-denied environments, the proposed system reconstructs the pedestrian trajectory solely from single-IMU measurements by coupling gait-derived stride information with attitude orientation inferred from quaternion data. This integration allows the recovery of continuous motion paths that preserve both horizontal direction and vertical displacement without relying on any external reference. Figure 10 presents the conceptual framework of the proposed pose-integrated 3D path reconstruction method, illustrating how stride length, heading direction, and posture state are fused through quaternion-based orientation coupling to generate a complete spatial trajectory of human motion.

In Figure 10, blue arrows indicate the step-to-step trajectory in the global frame, green arrows $R_{k}$ denote the heading/orientation vectors of each step, the black dashed arrow represents the vertical displacement $\Delta Z_{k}$, and gray arrows show the global X,Y,Z axes.

As shown in Figure 10, the proposed framework reconstructs a continuous 3D trajectory by combining stride estimation with pose orientation derived from the single-IMU quaternion data. Each step displacement is successively aligned with the body’s orientation to preserve both horizontal direction and vertical motion. The figure conceptually illustrates how stride length, heading, and elevation change are fused within a unified coordinate system, forming the basis for the analytical model detailed in the following subsections.

Previous studies have reported high accuracy in step count recognition. For instance, Luu et al. proposed a deep learning-based accelerometer step detection method [46], achieving recognition rates of 96–99% across different device conditions. Nevertheless, step count statistics alone are insufficient to support robust three-dimensional path estimation in complex environments. This limitation is particularly evident under GNSS-denied conditions, where the absence of stride length and posture modeling significantly constrains path reconstruction performance. To address these shortcomings, the present study incorporates *Z*-axis rhythm-driven three-state recognition together with a dynamic stride length estimation mechanism, thereby enhancing the adaptability and accuracy of agent trajectory reconstruction.

In this study, we propose a pose-integrated path reconstruction method capable of estimating the continuous 3D path of the human body without external position references. This method integrates stride estimation, pose orientation inference, and stair-walking state recognition, and applies a design concept based on “Stride Length + Orientation Direction” (SL + Orientation Vector) [47] to fuse gait parameters and pose information. This enables stable, real-time path reconstruction even in complex indoor environments.

#### 2.5.1. Two-Dimensional Planar Orientation Modeling (SL + Orientation Vector)

In the planar direction, the system reconstructs the 2D trajectory using a “stride length + orientation direction” approach. To achieve this, the current pose is converted into an orientation matrix through quaternion operations, and the first column vector of the matrix $R\in {\mathbb{R}}^{3\times 3}$ is adopted as the *X*-axis direction in the body coordinate system, that is:

(9) $${\vec{d}}_{k}^{xy}=R[:,0]$$

The amount of movement change in each gait step is determined jointly by the current stride length estimate $SL_{k}$ and the posture direction vector ${\vec{d}}_{k}^{xy}$, and its calculation formula is as follows:

(10) $$\left\{\begin{array}{c} x_{k}=x_{k-1}+SL_{k}\cdot d_{x,k}\\ y_{k}=y_{k-1}+SL_{k}\cdot d_{y,k} \end{array}\right.$$

Here, $d_{x,k}$ and $d_{y,k}$ represent the projected components of the direction vector on the horizontal plane.

#### 2.5.2. *Z*-Axis Step-Based Elevation Change Model

For the vertical position change $\Delta z_{k}$, the system makes a judgment based on the current stair walking status:

(11) $$\Delta z_{k}=\left\{\begin{array}{l} +h,\;if\;\;stair\_state_{k}=UP\_STAIRS\\ -h,\;if\;stair\_state_{k}=DOWN\_STAIRS\\ 0,\;\;\;\;if\;\;\;stair\_state_{k}=FLAT \end{array}\right.$$

h is computed in real time from the stride length $L_{k}$ and the walking posture angle $\theta_{k}$, and its calculation is given as follows:

(12) $$h=L_{k}\times sin(\theta_{k})$$

Here, $L_{k}$ denotes the currently estimated step length, and $\theta_{k}$ represents the inclination angle between the direction of motion and the ground in the body coordinate system, which is derived from the *Z*-axis component of the current attitude matrix $R_{b2n}$. The h value obtained in this manner preserves the simplicity of the computational structure while providing geometric adaptability to different staircase slopes, thereby ensuring both the stability of height-change estimation and its consistency with the actual environmental structure.

#### 2.5.3. Three-Dimensional Path Fusion Update

Finally, the accumulated 3D position is updated by integrating the planar displacement with the vertical increment:

(13) $${\vec{P}}_{k}={\vec{P}}_{k-1}+SL_{k}\times {\vec{d}}_{xy}^{k}+{\left[0,0,\;\Delta z_{k}\right]}^{T}$$

## 3. Results

### 3.1. Experimental Setup

To validate the effectiveness and generalizability of the proposed single-IMU-based 3D trajectory reconstruction framework, a series of controlled experiments were conducted under multiple representative walking scenarios. The experimental design aimed to evaluate both the accuracy and robustness of the system by replicating real-world gait conditions involving diverse motion patterns and structural variations. Particular emphasis was placed on testing the method’s stability during long-distance planar walking, repeated directional turns, and complex vertical movements such as stair climbing.

**Figure 11:**
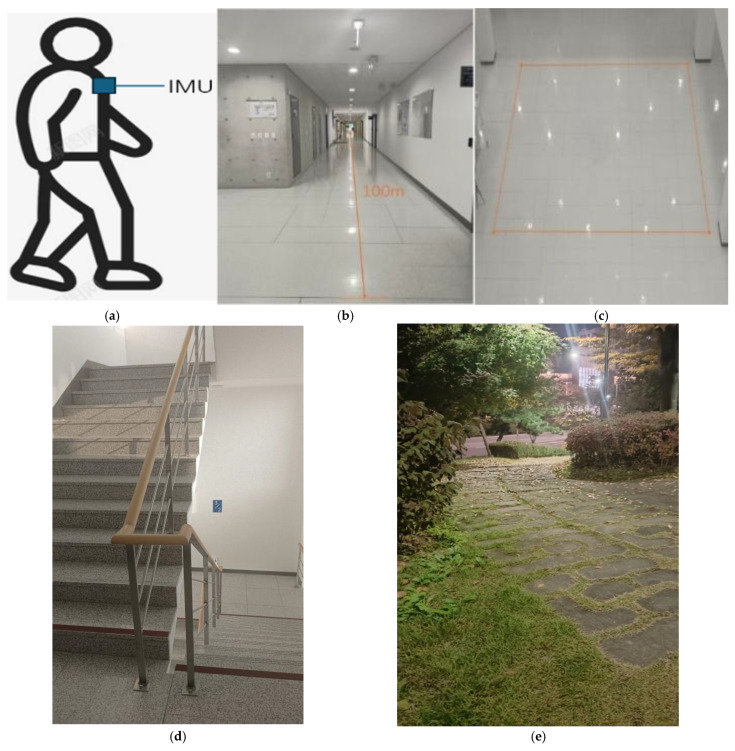
Experimental environment and IMU placement configuration. (**a**) Mounting position of the single inertial measurement unit (IMU) on the pedestrian’s chest, ensuring stable attitude estimation and consistent orientation tracking; (**b**) indoor corridor environment for straight-walking experiments, with a path length of approximately 100 m; (**c**) indoor rectangular path designed for planar turning tests, including segments with multiple directional changes; (**d**) stair-climbing environment used to evaluate vertical displacement estimation and gait-state adaptability across multiple floors; (**e**) outdoor sloped path employed to assess the robustness of the proposed framework under uneven-ground conditions.

This study systematically evaluated the proposed single-IMU-based 3D path reconstruction method across three representative walking scenarios to verify positional accuracy and robustness in complex environments. As Figure 11 shown, the test scenarios included: (1) straight walking along an indoor corridor, (2) planar square-turning motion involving multiple directional changes, (3) stair climbing and descending between multiple floors, (4) a crowded-corridor scenario involving passive avoidance and minor detours, and (5) outdoor slope walking on uneven ground. These experiments collectively cover the fundamental movement patterns and environmental structures commonly encountered in natural pedestrian motion.

The system acquires tri-axial accelerometer, gyroscope, and magnetometer signals, along with posture quaternions, at a sampling rate of 200 Hz. Data are transmitted to the host in real time via a serial link. Upon reception, the data are re-timestamped, aligned to a unified time base, and incrementally updated using a sliding window. This process establishes a stable processing pipeline for subsequent modeling and analysis.

The experimental protocol encompassed five representative path types designed to evaluate different aspects of the proposed framework. In the indoor straight-walking scenario, the pedestrian agent walked approximately 100 m along a corridor and returned along the same route. This test verified the system’s ability to suppress accumulated errors on flat ground, while robustness to velocity perturbations was examined by varying walking speeds. The planar square-turning experiment involved walking along a rectangular loop with four orthogonal corners, traversed in both clockwise and counterclockwise directions. This configuration enabled assessment of directional perception, rotation continuity, and planar trajectory reconstruction accuracy. The stair-climbing scenario consisted of repeated ascent and descent between multiple floors, validating the system’s capacity to estimate inter-floor height variations and adapt to changes in gait rhythm and stair geometry. To further assess robustness in more dynamic conditions, a crowded-corridor test was conducted, in which participants performed passive avoidance and minor detours to simulate real-world pedestrian interactions. This experiment examined the system’s stability in gait state recognition and trajectory continuity under transient disturbances. Finally, the outdoor slope-walking test evaluated performance on uneven terrain and under natural lighting conditions, demonstrating the system’s adaptability to unstructured environments beyond controlled indoor settings.

### 3.2. Experimental Results and Error Analysis

To further assess the overall performance of the proposed single-IMU 3D trajectory reconstruction framework, the reconstructed trajectories were compared with reference paths obtained under controlled indoor and semi-outdoor conditions. The evaluation covered five representative motion scenarios—straight walking, square turning, stair climbing, crowded-corridor avoidance, and outdoor slope walking—representing both planar and vertical gait patterns as well as structured and unstructured environments.

**Figure 12:**
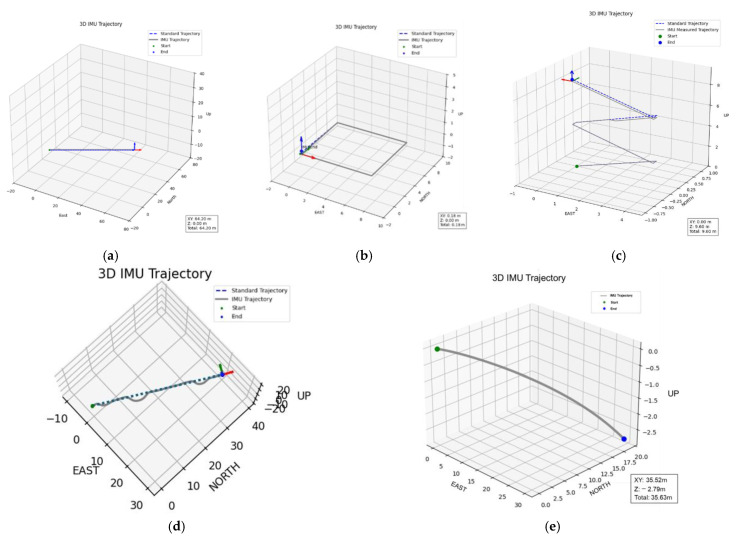
Reconstructed 3D trajectories under four representative walking scenarios using the proposed single-IMU framework. In (**a**–**e**), the gray solid line represents the IMU-estimated trajectory, the blue dashed line denotes the reference path, the green marker indicates the start point, and the blue marker indicates the end point: (**a**) Straight walking path; (**b**) Square-turning path; (**c**) Stair-climbing scenario; (**d**) Crowded-corridor scenario with passive avoidance and minor detours; (**e**) outdoor sloped path. The red, green, and blue arrows denote the X, Y, and Z axes, respectively.

As shown in Figure 12, the proposed system successfully reproduces stable and geometrically consistent trajectories across all conditions. The reconstructed paths closely align with the reference trajectories, demonstrating strong adaptability, directional stability, and scale fidelity even under gait disturbances and environmental variations [1,2,3,4,5,6].

Each walking scenario was repeated approximately fifty times under identical experimental conditions to ensure statistical reliability and reproducibility. The IMU signals were sampled at 200 Hz. For both the flat-ground and stair-climbing experiments, tests were conducted over multiple path lengths and elevation levels, and the reported trajectories and numerical results represent the averaged outcomes across all repetitions.

In the context of real-time trajectory reconstruction, the proposed system continuously estimates stride length and walking direction in an online manner. As shown in Figure 12, the reconstructed trajectories exhibit stable geometric continuity and strong agreement with the reference paths across all representative scenarios. In the straight-walking test, the reconstructed trajectory aligns closely with the reference path, maintaining a mean horizontal error below 3%, thereby validating the linear scale consistency of the proposed dynamic stride-length model. In the square-turning test, the system accurately captured corner transitions and restored orientation after each turn, confirming the reliability of attitude inference during rotational motion. In the stair-climbing scenario, the reconstructed 3D trajectory successfully represented vertical displacement between floors, with an average height estimation error of approximately 3%. In the crowded-corridor experiment, small lateral deviations caused by passive avoidance and detour behavior were effectively corrected as the system reestablished the main walking direction, demonstrating high robustness under transient gait disturbances. Finally, in the outdoor slope-walking test, the proposed framework maintained directional and scale stability under uneven terrain, further validating its applicability in unstructured environments.

### 3.3. Comparison with Existing IMU-Based PDR Methods

To further evaluate the performance of the proposed method, its experimental accuracy was compared with representative IMU-based pedestrian dead reckoning (PDR) approaches reported in the literature. Table 1 presents a concise summary of the average horizontal and vertical errors achieved by different algorithms under similar conditions. Traditional ZUPT-based systems [35,36] typically maintain planar accuracy within 3–5% over a 100 m path but exhibit significant drift accumulation during vertical transitions. Adaptive stride-length models [37,38] improve horizontal stability, yet their height estimation remains less precise (≈4%) and requires user-dependent calibration. Deep-learning-assisted IMU methods [39,40,41,42] achieve mean horizontal errors below 2%, but at the cost of heavy computation and large training datasets.

In contrast, the proposed single-IMU mechanism maintains mean planar errors below 3% and vertical errors under 2% across both flat and stair environments, without any external references or learning models.

This demonstrates that the unified coupling of stride adaptation, *Z*-axis rhythm recognition, and quaternion-based pose projection provides a practical trade-off between accuracy, robustness, and computational efficiency, outperforming classical model-based systems and approaching the accuracy of learning-based frameworks.

While multi-sensor fusion approaches achieve the best numerical accuracy, the proposed single-IMU framework attains near-equivalent performance with dramatically reduced complexity and dependency, offering a practical and scalable alternative for GNSS-denied environments.

## 4. Discussion

This study proposes a 3D path estimation method based on a single IMU to meet the demand for lightweight positioning in environments where GNSS signals are unavailable. The proposed mechanism integrates stationary state recognition, *Z*-axis extremum-based gait rhythm modeling, and real-time stride length estimation to construct a system capable of maintaining orientation accuracy, scale adaptability, and drift suppression. Experimental evaluations demonstrate that the method can reconstruct continuous, stable, and highly accurate 3D trajectories.

Results show consistent performance across diverse scenarios, including stationary states, constant-velocity walking, variable-speed turns, and stair climbing. The system further satisfies real-time reconstruction requirements by recognizing walking states within milliseconds and computing paths with centimeter-level precision.

- Planar Path Accuracy: On a 100 m horizontal path, the mean position error remained below 3%, surpassing practical standards for general applications.
- Vertical Accuracy: In stair-climbing scenarios up to 10 floors, the mean error rate was approximately 3%, validating the reliability of vertical displacement estimation.

In summary, the originality of this research is reflected in its architecture that unifies gait state recognition, adaptive stride modulation, and attitude-based displacement projection into an integrated, learning-free pipeline. This combination enables real-time 3D reconstruction without external sensors or calibration, thereby establishing a novel framework for low-cost, high-precision positioning. The IMU-based path reconstruction mechanism developed in this study exhibits robust continuity, stable navigation, and strong adaptability to complex environments without requiring support from external positioning systems. These features make it highly relevant for practical scenarios such as indoor navigation in large facilities, disaster relief operations, and underground space exploration, highlighting both its engineering significance and scalability.

The mechanism adopts a dedicated Pedestrian Dead Reckoning (PDR) architecture, enabling direct deployment on lightweight platforms including smartphones, wearable devices, and smart glasses. Such a design broadens its applicability to tasks such as large-scale indoor guidance, personnel localization in emergency situations, and walking assistance for the elderly and visually impaired. Its lightweight structure further ensures long-term operation in mobile environments with constrained battery capacity. By removing the dependency on external infrastructures such as Wi-Fi fingerprinting or UWB anchors, the approach reduces installation and maintenance costs while offering inherent advantages in privacy protection.

Future research will focus on extending the mechanism through calibration strategies that dynamically adapt to agent-specific characteristics in real time and the integration of multidimensional gait metrics—such as stride length, gait cycle, and posture variations—to construct personalized agent models. In addition, systematic evaluations in crowded and multi-story environments, as well as investigations targeting diverse user groups including elderly individuals, children, and people with disabilities, are expected to further validate its robustness. Ultimately, this lightweight mechanism has the potential to evolve into a high-precision, PDR-centered navigation technology and to serve as a key enabler for emerging urban applications such as smart city management, digital twin-based indoor monitoring, and disaster-response robotics.

Moreover, attention will be given to the statistical and environmental factors influencing IMU-based positioning accuracy. Although the deterministic framework proposed in this study effectively constrains short-term drift through denoising, adaptive thresholding, and stationary correction, certain stochastic effects—such as sensor bias instability, external vibration, and model approximation errors—remain partially unmodeled. Future work will therefore incorporate stochastic bias modeling, covariance propagation, and probabilistic error characterization to analytically describe uncertainty evolution. These improvements are expected to further enhance the system’s robustness and universal applicability across diverse IMU hardware and operating conditions, providing a pathway toward a statistically grounded, hardware-independent localization framework.

Ultimately, this lightweight mechanism has the potential to evolve into a high-precision, PDR-centered navigation technology and to serve as a key enabler for emerging urban applications such as smart city management, digital twin-based indoor monitoring, and disaster-response robotics.

## Figures and Tables

**Table 1:** Comparative analysis of representative PDR methods and the proposed physics-driven IMU-only framework.

Method	Sensor Setup	Learning/Fusion	Planar Error
ZUPT-PDR [35,36]	Foot-mounted IMU	None	3~5%
Adaptive Stride Model [37,38]	Body IMU	None	≈3%
Multi-Sensor Fusion [12,13,14,15,16,17,18,19,20,21]	IMU + GNSS/UWB/LiDAR	EKF/FGO Fusion	<1%
Deep-Learning PDR [39,40,41,42]	IMU + DL	CNN/LSTM/GRU	1~2%
Proposed Method	Single IMU (X-IMU3)	None	<3%

## Data Availability

The original contributions presented in the study are included in the article; further inquiries can be directed to the corresponding author.
